# Prevalence and characteristics of coronary artery anomalies (CAAS) in 3016 symptomatic adult participants undergoing coronary computed tomography angiography (CCTA): A single-center retrospective study in Iran

**DOI:** 10.34172/jcvtr.2023.32860

**Published:** 2023-12-30

**Authors:** Abbas Andishmand, Hossein Montazerghaem, Ali Pedarzadeh, Hamid Reza Varastehravan, Hamidreza Mohammadi, Reza Nafisi Moghadam, Marzieh Azimizadeh, Mohammad Hossein Ahrar, Abdolrahim Khezri, Mohsen Andishmand

**Affiliations:** ^1^Yazd Cardiovascular Research Center, Non-communicable Diseases Research Institute, Shahid Sadoughi University of Medical Sciences, Yazd, Iran; ^2^Cardiovascular Research Center, Hormozgan University of Medical Sciences, Bandar Abbas, Iran; ^3^Department of Cardiology, Shahid Sadoughi Hospital, Shahid Sadoughi University of Medical Sciences, Yazd, Iran; ^4^Department of Radiology, Shahid Sadoughi Hospital, Shahid Sadoughi University of Medical Sciences, Yazd, Iran

**Keywords:** Computed tomography, Computed tomography angiography, Coronary artery anomalies, Prevalence, Coronary vessels

## Abstract

**Introduction:**

Coronary artery anomalies (CAAs) are associated with an increased risk of cardiovascular events, including sudden cardiac death, especially in young people. A different prevalence has been reported based on the USED diagnostic modality. This study aimed to determine the prevalence and type of these anomalies using coronary computed tomography angiography (CCTA).

**Methods:**

This single-center retrospective study was performed on 3016 consecutive cases who underwent CCTA for cardiac symptoms from March 2015 to August 2020 and the prevalence and types of CAAs were evaluated.

**Results:**

38 cases (overall prevalence of 1.26%) including 21 men (55.3%) and 17 women (44.7%) were retrospectively diagnosed with CAAs. The most common anomalies were the Anomalous origin of LCX from the right coronary sinus (11 cases, 28.9%), Anomalous origin of RCA from the left coronary sinus (11 cases, 28.9%), and Anomalous origin of LM from the right coronary sinus (6 cases, 15.8%). There was no difference in the prevalence of CAAs in terms of patient’s gender (*P* value=0.16) and age (*P* value=0.61).

**Conclusion:**

The prevalence of CAAs among patients who underwent CCTA was 1.26%. The most common anomalies observed were the anomalous origin of the LCX arising from the right coronary sinus, the anomalous origin of the RCA arising from the left coronary sinus, and the anomalous origin of the LM arising from the right coronary sinus. These findings emphasize the importance of CCTA in detecting and characterizing coronary artery anomalies, which may have clinical implications for patient management and treatment decisions.

## Introduction

 Although the prevalence of coronary anomalies in the community is low, it is an important cause of cardiovascular events, including sudden cardiac death, especially in young people. In the United States, the rate of sudden cardiac death because of coronary artery anomalies (CAAs) is reported at 5% to 31% ^1-3^. Before new imaging modalities, coronary angiography was the only method of diagnosing CAAs before death. Although angiography is the standard method for diagnosing coronary anomalies, it has been gradually replaced by new imaging techniques because of its invasiveness and potentially fatal complications ^4,5^. Depending on the modality used and the study population, different values have been reported as an estimate of the prevalence of CAAs. According to angiographic findings, the prevalence of coronary anomalies is 0.9%-1.3%, while with CT angiography it is reported to be 0.43%-5.79% ^4-6^. This study aimed to determine the prevalence of CAAs in adults who underwent Coronary Computed Tomography Angiography (CCTA) because of cardiac symptoms.

## Materials and Methods

###  Patient selection

 This cross-sectional retrospective study was conducted on 3016 consecutive patients with chest pain who underwent CCTA at Shahid Sadoughi hospital, Yazd, Iran, from March 2015 to August 2020. The patient was not charged any fees outside of the treatment, and written informed consent was obtained from all patients. Indications for CCTA included chest pain, angina pectoris, preoperative assessment for non-coronary surgery, scanning for coronary artery disease, and assessing the efficacy of bypass grafts or stents. Patients with uncontrolled heart rates, severe arrhythmia, and severe coronary calcification were excluded.

###  Preparation

 Before the scanning, all patients were given 0.4 mg of sublingual nitroglycerin and, if necessary, 50-200 mg of metoprolol orally to get the heart rate less than 65 beats per minute at the time of scanning for optimal image quality.

###  Procedure

 Using prospective electrocardiographic triggering (for most of the cases) or retrospective electrocardiographic gating acquisitions, CCTA was performed on a 128-slice CT scanner (Somaton Definition Edge, Siemens Healthineers, Erlangen, Germany) during a breath hold. Iodixanol (Visipaque 320, 320 mg/ml) was injected via an antecubital vein followed by a 50 ml saline solution at a rate of 6 mL/s. The volume and velocity of the flow were proportional to the body weight. All data were processed and analyzed using a dedicated workstation for image reconstruction and evaluation. Analysis and interpretation of the images were performed using the cardiac phase with the best axial, sagittal, and coronal MPR, Curved MPR, MIP, and 3D volume rendering reconstructions. CAAs were classified into anomalies of origin and course, intrinsic coronary arterial anatomy, and termination, and evaluated by a cardiovascular radiologist ^7^. Myocardial bridging was considered a normal variant and not included in the current study. Coronary ectasia also were not included as anomalies in this study.

###  Statistical Analysis

 Statistical analysis was performed using SPSS software version 19 (SPSS Inc., Chicago, IL, USA). Quantitative and qualitative variables were shown as mean ± standard deviation or frequency and percentage, respectively. Continuous variables and categorical variables were analyzed by T-test and chi-squared test, respectively. A *P* value of less than 0.05 was considered statistically significant.

## Results

**Figure 1:**
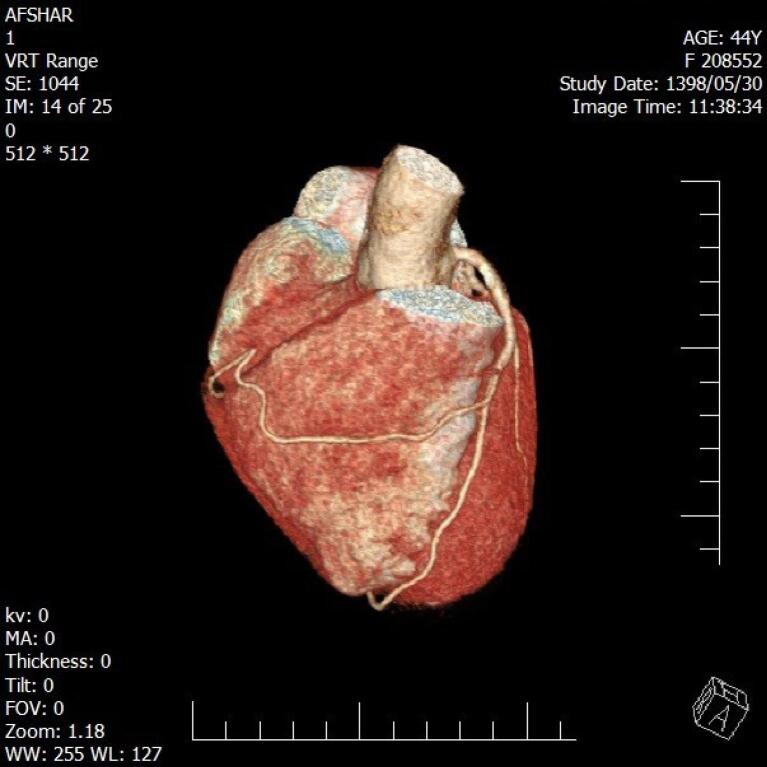
A 44-year old man with a history of exertional chest pain and CCTA showing the origin of the RCA artery from the LAD artery

**Figure 2:**
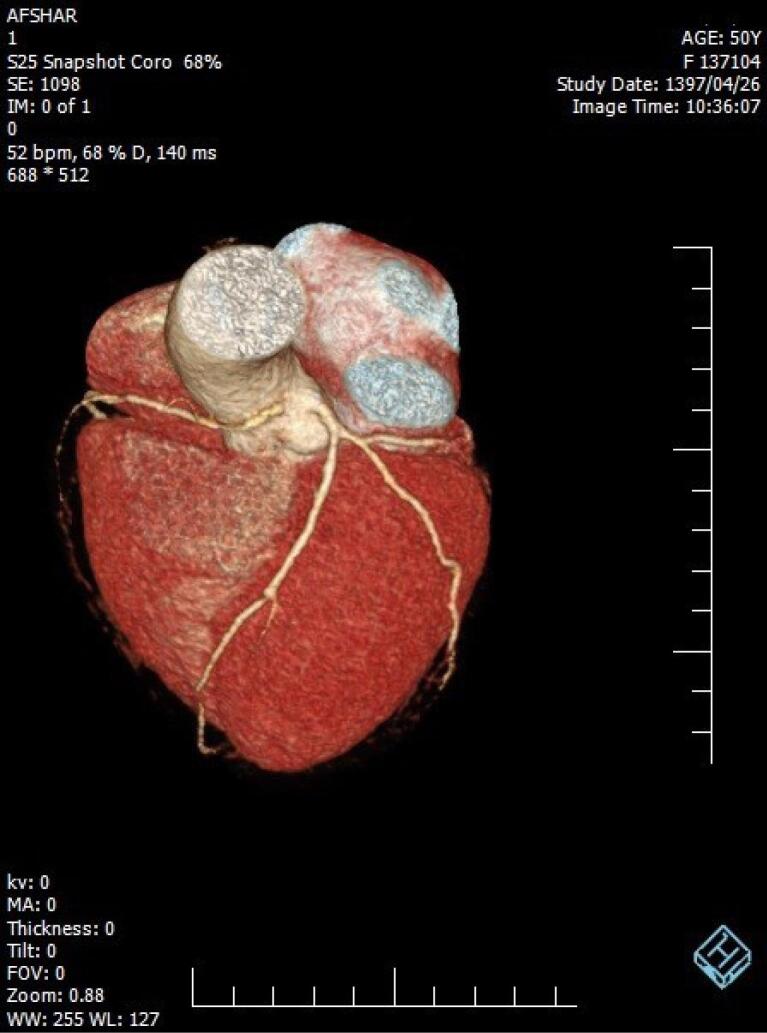
A 50-year old man with a history of chest pain and CCTA showing the origin of the RCA artery from the left coronary sinus

**Figure 3:**
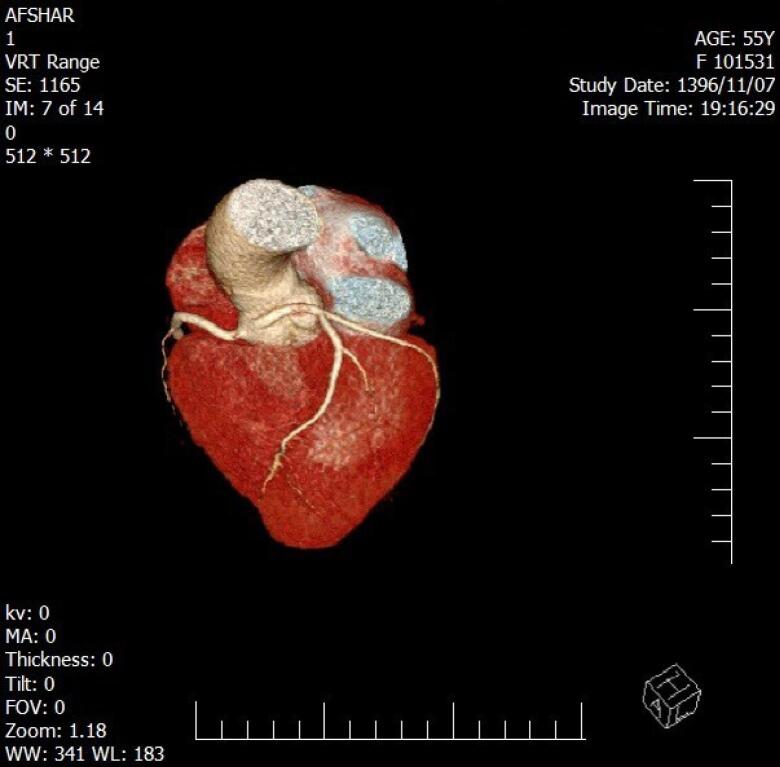
A 55-year old man with a history of chest pain and non diagnostic Exercise test and CCTA showing the origin of the left coronary artery from the right coronary sinus

**Figure 4:**
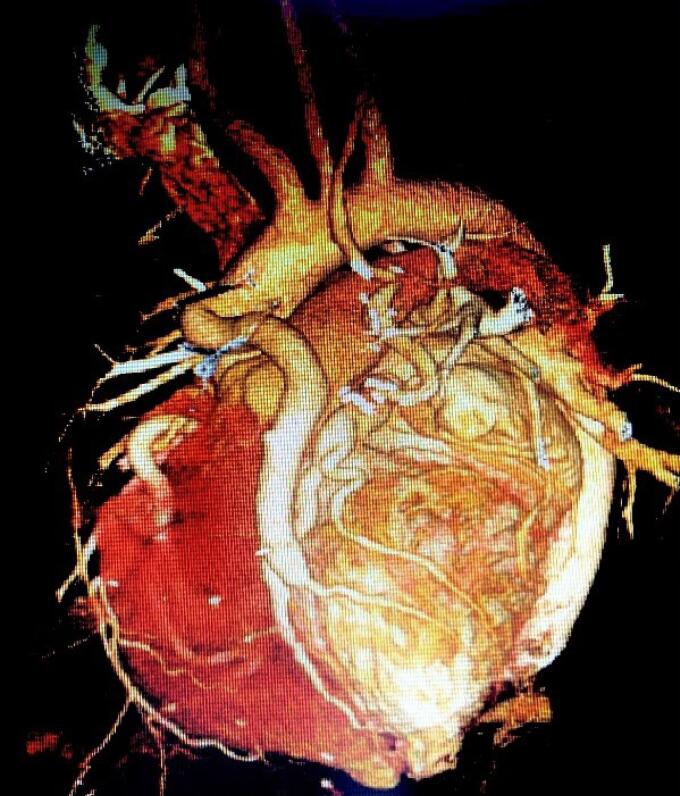
A 31-year old female with a history of exertional dyspnea and CABG surgery and CCTA showing the origin of the LM from PA (ALCAPA)

 During five years, among 3016 participants, a total of 38 individuals (overall prevalence of 1.26%) including 21 men (55.3%) and 17 women (44.7%) were diagnosed with coronary artery anomalies. The mean age was 54.82 ± 12.52 years. Chest pain (52.7%) appeared most often and was closely followed by shortness of breath (36.8%) (Table 1). The most common anomalies were the Anomalous origin of LCX from the right coronary sinus (11 cases, 28.9%), Anomalous origin of RCA from the left coronary sinus (11 cases, 28.9%), and Anomalous origin of LM from the right coronary sinus (6 cases, 15.8%). Other anomalies are shown in Table 2. There was no difference in the prevalence of CAAs in terms of patients’ gender and age (*P *value = 0.16 and *P *value = 0.612 respectively). Clinical variables, including symptoms and comorbidities, did not show a statistically significant relationship with coronary artery anomalies (*P* > 0.05). Figures 1-4 show some cases of coronary anomalies that we have found.

**Table 1:** Clinical characteristics

**Gender, N (%)**	
Male	21 (55.3%)
Female	17 (44.7%)
Age (Mean ± Standard Deviation)	54.82 ± 12.52
**Sign & Symptom, N (%)**	
Chest Pain	20 (52.7%)
Shortness Of Breath	14 (36.8%)
Palpitations	7 (18.4 %)
Syncope	5 (13.2%)
**Comorbidities, N (%)**	
Hypertension	18 (47.4%)
Diabetes Mellitus	10 (26.3%)
Hypothyroidism	1 (2.6%)

**Table 2:** Prevalence coronary artery anomalies (CAAs) in population undergoing coronary CT angiography

**Type of Anomaly**	**Number of cases**	**Percentage (%) among total anomalies**	**Incidence of Anomaly (%)**
Anomalous of origination and distribution			
LCX arising from right coronary sinus	11	28.94	0.364
RCA arising from the left coronary sinus	11	28.94	0.364
LM arising from right coronary sinus	6	15.78	0.199
LM arising from PA	1	2.63	0.033
LM from posterior Valsalva sinus	1	2.63	0.033
High take off RCA	4	10.52	0.132
Absent LM	2	5.26	0.066
Absent LCX	2	5.26	0.066
Total coronary anomalies	38	100	1.26
Total coronary CT angiography	3016		

LM: left main, LCX: left circumflex artery, RCA: right coronary artery, PA: pulmonary artery

## Discussion

 CAAs are usually thought to be very rare. CT coronary angiography is an accurate diagnosis tool for detecting coronary anomalies and studies have shown its superiority over invasive coronary angiography. Only 53% of cases of coronary artery abnormalities diagnosed with CCTA are detected on invasive coronary angiography ^8^. Therefore, the reason for the different statistics values of the prevalence of CAAs is the imaging method used. With the use of CCTA, a higher prevalence of anomalies has been reported compared to invasive coronary angiography and, CCTA is more sensitive to the diagnosis of CAAs ^9,10^. In a study of 126,595 patients undergoing invasive coronary angiography, the prevalence of coronary artery anomaly was estimated to be 1.3% ^6^. As mentioned earlier, in studies using CCTA, the prevalence of coronary artery abnormalities has been reported to be 0.43% -5.79% ^10-13^. The reasons for the differences in the estimated prevalence values between the different imaging techniques, and also with the same imaging method used, are because of ambiguity in the definition of CAAs and the effect of referral bias on the study results ^14^. In the present study, which was performed on 3016 symptomatic patients with suspected coronary heart disease, the CCTA estimated the overall prevalence of 1.26% for CAAs. According to Angelini’s classification which is considered the most comprehensive classification of CAAs, Anomalous origin of the coronary artery from the opposite sinus (ACAOS) was the most prevalent type of coronary artery anomaly found in our study with an estimated prevalence of 829 per 100 000 (28 cases, 73.4%). In one study using CCTA, the prevalence of ACAOS was reported to be 0.84% ^15^. The clinical importance of ACAOS is its association with the potential risk of sudden cardiac death. In a study to determine the cause of the sudden death of 134 athletes, ACAOS was the second leading cause of acer hypertrophic cardiomyopathy (34% and 13%, respectively) ^16^. Another study of military recruits found that 33% of the causes of cardiac deaths were ACAOS and only 52% were symptomatic before the occurrence of sudden cardiac death ^17^. Most of these tragic deaths occur during or immediately acer exercises ^7^. This highlights the importance of discovering ACAOS, especially in high-risk groups such as athletes and important occupations that apply to people’s lives in society. Single coronary arteries, anomalous origin of the left coronary artery from pulmonary artery (ALCAPA) and coronary artery fistulas are other types of CAAs that might be associated with cardiac events and these anomalies are all best diagnosed with cardiac CT. Therefore, CCTA can be considered as an accurate, rapid, and relatively safe method for identifying these high-risk groups, even if they are asymptomatic. An uncommon coronary artery anomaly that we found was a case of ALCAPA (Anomalous left coronary artery from the pulmonary artery) with a prevalence of 33.2 per 100 000 population. The incidence of ALCAPA in live births is 1 in 100 000 and is 95% isolated. If left untreated, it can lead to death early in life ^18^. Another relatively rare but important coronary artery anomaly is High take off the ostium of the RCA. In this anomaly, the right coronary artery originates above the right sinus of Valsalva. Its prevalence is 0.019 to 0.17% in autopsy series and 0.25% in invasive coronary angiography studies. Although it is mostly an incidental finding, it has been described in the literature as the cause of angina pectoris, myocardial infarction, and sudden cardiac death ^19^. In our study, the prevalence of this anomaly was estimated at 133 per 100 000 population of those undergoing CCTA. In summary, we could detect serious and inherently fatal CAAs in symptomatic patients undergoing CCTA. Finding these important anomalies with CCTA can be the beginning of future decisions for basic therapeutic interventions, including surgery, as well as preventative recommendations with lifestyle changes, avoidance of strenuous and competitive sports activities, and periodic clinical visits if needed. Our cross-sectional study was performed only on symptomatic patients and therefore, many people who were apparently healthy but may have serious CAAs were not included. Referral bias is present for all studies and screening the entire population to find these anomalies, which in 80% of cases are benign, is not cost-effective.

## Conclusion

 The prevalence of coronary artery anomalies (CAAs) among patients undergoing coronary CT angiography (CCTA) was found to be 1.26%. The most common anomalies observed were the anomalous origin of the LCX arising from the right coronary sinus, the anomalous origin of the RCA arising from the left coronary sinus, and the anomalous origin of the LM arising from the right coronary sinus. These findings emphasize the importance of CCTA in detecting and characterizing coronary artery anomalies, which may have clinical implications for patient management and treatment decisions. However, further research is necessary to explore the clinical significance and long-term outcomes associated with these anomalies. Additionally, larger-scale studies involving diverse populations are needed to validate our results and provide a more comprehensive understanding of the prevalence and characteristics of CAAs.

## Acknowledgments

 We would like to thank Mr. Mahdi Jafari, the chief technologist of the CT scan department of Afshar Hospital in Yazd, who provided us with the access to patients’ information.

## Competing Interests

 There is no conflict of interest for authors involved in this study.

## Ethical Approval

 This study was conducted after the approval of the local ethics committee of Shahid Sadoughi University of medical sciences (IR.SSU.MEDICINE.REC.1398.276).

## Funding

 None.
